# 1 km-resolution gridded dataset of phosphorus rate for rice wheat and maize in China over 2004–2016

**DOI:** 10.1038/s41597-023-02283-z

**Published:** 2023-06-07

**Authors:** Wenmeng Zhang, Tianyi Zhang, Xiaoguang Yang

**Affiliations:** 1 College of Resources and Environmental Sciences, China Agricultural University, Beijing, China; 2 State Key Laboratory of Atmospheric Boundary Layer Physics and Atmospheric Chemistry, Institute of Atmospheric Physics, Chinese Academy of Sciences, Beijing, China; 3 Collaborative Innovation Center on Forecast and Evaluation of Meteorological Disasters, Nanjing University of Information Science & Technology, Nanjing, China

**Keywords:** Environmental impact, Sustainability

## Abstract

Crop-specific, high-resolution phosphorus rate information is essential for sustainable agricultural fertilizer management in China. However, substantial uncertainties exist in the current phosphorus fertilizer dataset because of only coarse national statistics used in dataset development and no crop-specific information provided. This study harmonized provincial and county-level phosphorus and component fertilizer statistics and crop distribution data to generate 1 km gridded maps of phosphorus rate for rice, wheat and maize in the years of 2004–2016 (CN-P). CN-P provides a comparable estimate on phosphorus rate for each crop over 2004**–**2016, and demonstrates an improved spatial heterogeneity. Existing dataset developed using national statistics tends to smooth out the variability within country and significantly underestimates actual phosphorus rate. CN-P shows that, during 2004–2016, wheat received the most phosphorus rate (8.7 g P_2_O_5_ m^−2^), while maize showed the rapidest increasing trend (2.36% yr^−1^). The CN-P dataset has the potential to be widely applied in modeling studies on sustainable agricultural fertilizer management strategies and phosphorus pollution.

## Background & Summary

Phosphorus plays a vital role in growing crops^1,2^ and meeting the food demands^3–5^. China is the largest producing and consuming country of phosphorus fertilizer^6^. In 2020, there is around 13 million tons phosphorus fertilizers produced in China, and approximately 9 million tons were used in agriculture, which accounts for 20.6% of the global phosphorus fertilizer consumption^7^. Since 1980, the total phosphorus fertilizer application has grown, and substantial phosphorus fertilizer demands were projected in 2050 due to growing populations in China^4^.

Overuse of phosphorus fertilizer results in a series of environmental problems^8^. Currently, there are twenty provinces are subjecting to different level phosphorus pollution^9^. Soil available phosphorus was found increased from 17.09 mg L^−1^ in the 1990s to 33.28 mg L^−1^ in the 2000s in China^10^; and 48 Tg phosphorus has leached to water bodies in the past 60 years^3,11^. As a consequence, eutrophication was found in 23.6% major lakes in China, and 4.5% were moderately eutrophic, and 0.9% were severely eutrophic^12^. Therefore, sustainable phosphorus fertilizer management is crucial to both food security and environmental conservation.

To investigate the efficient phosphorus fertilizer management, it is critical to understand the state of agricultural phosphorus rate. Previous studies have been examining the historical phosphorus footprint^6^, phosphorus budge^1^ and phosphorus losses^3^. These analyses are typically based on the national-scale phosphorus statistics from FAOSTAT^7^ and IFASTAT^13^. A gridded agricultural phosphorus dataset was developed by Lu and Tian^14^, in which they obtained the gridded phosphorus consumption by multiplying the national-scale phosphorus rate statistics with gridded cropland data. Despite widely used, there are three main deficiencies in the existing dataset: (1) The most-widely available phosphorus statistic is national-scale. This is a very coarse data, and tends to lump the spatial heterogeneities within country; (2) Data source of phosphorus rate was from statistics department and cropland data was obtained from remote sensing method in the data product. Direct composite of the two kinds of data may raise the inconsistency issue of statistical caliber; (3) The current gridded phosphorus dataset only provides the total agricultural phosphorus information, rather than crop-specific. The lack of crop-specific data leads to the difficulty to separate phosphorus fertilizer by various crops and growing seasons in modeling analysis.

To overcome these deficiencies, we constructed a new gridded phosphorus rate dataset, which was referred to as CN-P. CN-P is 1 km-resolution gridded dataset of phosphorus rate for three staple food crops (i.e. rice, wheat and maize) in China over 2004–2016. This contains gridded maps of phosphorus rate in each year by each crop. In data construction, we employed the county-level statistic, which is the finest agricultural statistics in China. After validation, we found that CN-P demonstrates a comparable crop-specific phosphorus rate with previous farmer’s surveys, and provides an improved spatially-explicit distribution relative to previous state-of-the-art dataset when compared for individual year. We found that only using national statistics in previous gridded phosphorus dataset tends to smooth out the spatial variability and significantly underestimates agricultural phosphorus rate.

## Data and Methods

### Data sources

Multiple data were used to construct CN-P (Table 1). Statistical yearbooks, “Cost-benefit statistics of agricultural products”, provide the provincial historical data on crop-specific phosphorus and component fertilizer rate over 2004–2016^15–27^. Historical provincial crop-specific growing areas, total agricultural growing areas and total phosphorus and component fertilizer consumptions were downloaded from National Bureau of Statistics of China^28^. The above provincial data includes 31 provinces in the mainland of China, excluding Hongkong, Macau and Taiwan. The county-level statistics were based on “China County Statistical Yearbook”^29–41^, compiled by Chinese Academy of Agricultural Sciences. Note that the county-level phosphorus and component fertilizer consumption statistics only report the total fertilizer consumption, but not crop-specific. The county-level growing areas include both statistics of crop-specific and total agricultural growing areas in 2267 counties. The crop distribution data are based on 1 km gridded crop growing area distribution maps for rice, wheat and maize between 2004 and 2016 obtained from National Science & Technology Infrastructure^42^.

**Table 1:** Summary of statistics and data used in the study.

Dataset	Spatial resolution	Variables	Data sources
Cost-benefit Statistics of Agricultural Products	Province	Phosphorus and component fertilizer rate by crops	National Development and Reform Commission of China^15–27^
China Statistics by Province	Province	Total phosphorus and component fertilizer consumptions; Crop-specific growing areas; Total agricultural growing areas	National Bureau of Statistics of China^28^
China County Statistical Yearbooks	County	Total phosphorus and component fertilizer consumptions; Crop-specific growing areas; Total agricultural growing areas	Rural Socioeconomic Survey Team^29–41^
1 km Gridded Crop Growing Area Distribution	1 km resolution	Growing area distribution of rice, wheat and maize	Luo and Zhang^42^

### Methods

**Fig. 1:**
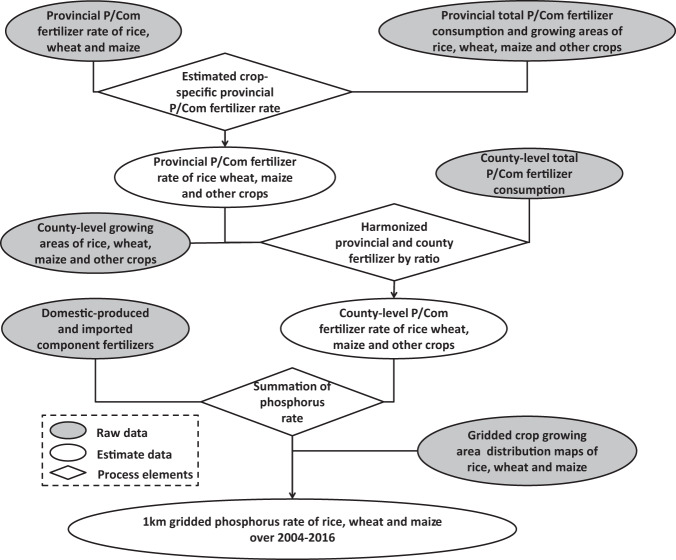
Diagram for processing spatially explicit time series of phosphorus rate of rice, wheat and maize in China over 2004–2016. “P” refers to phosphorus fertilizers and “Com” refers to Component fertilizers.

Figure 1 illustrates the steps of data processing, in which we harmonized above provincial/county-level phosphorus and component fertilizer statistics and crop distribution data to generate 1 km gridded maps of phosphorus rate for rice, wheat and maize in the years of 2004–2016.

#### Crop-specific phosphorus/component fertilizer rate in provinces

We firstly multiplied the provincial phosphorus and component fertilizer rate of rice, wheat and maize with their growing areas to generate crop-specific phosphorus and component fertilizer consumption at each province in each year. After that, subtracting above three crops from the total phosphorus and component fertilizer consumption at each province can produce the provincial phosphorus and component fertilizer consumption of other crops. We then divided the phosphorus and component fertilizer consumption of other crops by growing areas of other crops to obtain the phosphorus and component fertilizer rate of other crops at each province in each year. Following above steps, we generated the phosphorus and component fertilizer rate of rice, wheat, maize and other crops at each province over 2004–2016.

#### Harmonizing the crop-specific statistics of provinces with county statistics

Using the county statistics, we firstly calculated the growing area of rice, wheat, maize and other crops in each county-year combination using the same method for provincial data in the above step. For the missing data, cubic spine interpolation method was employed to gap-fill when data were unavailable in less than 3 consecutive years. To harmonize the provincial and county-level statistics, we did not simply multiply the two statistics directly, as did in previous dataset^14^. Instead, we calculated the ratio of crop-specific phosphorus/component fertilizer consumption to county total as Eq. (1). Then, the phosphorus/component fertilizer consumption for crop-specific in each county-year combination can be estimated by multiplying the county-level total phosphorus/component fertilizer consumption statistics with above ratios as Eq. (2). Finally, these fertilizer consumptions divided by the crop-specific county-level growing area statistics can produce the phosphorus /component fertilizer rate of rice, wheat and maize at each county in each year as Eq. (3). This practice enables us avoiding the potential inconsistency problem of statistical calibers compared to the direct computation method in early study^14^, and maintaining the spatial heterogeneities between counties. Further comparison can be seen in the technical validation section.

1 $${{\rm{Rat}}}_{i,j,y}=\frac{{{\rm{FR}}}_{i,p,y}\times {{\rm{A}}}_{i,j,y}}{{\sum }_{i}{{\rm{FR}}}_{i,p,y}\times {{\rm{A}}}_{i,j,y}}$$

2 $${{\rm{FC}}}_{i,j,y}={{\rm{Rat}}}_{i,j,y}\times {{\rm{FC}}}_{j,y}$$

3 $${{\rm{FR}}}_{i,j,y}=\frac{{{\rm{FC}}}_{i,j,y}}{{{\rm{A}}}_{i,j,y}}$$

where FR_*i, p, y*_ is phosphorus /component fertilizer rate for crop type *i*, at province *p*, in year *y*; A_*i, j, y*_ is growing area statistics for crop type *i*, at county *j* located in the province *p*, in year *y*; Rat_*i, j, y*_ is the ratio of phosphorus /component fertilizer consumption for crop type *i* to the summed fertilizer consumption $\left({\sum }_{i}{{\rm{FR}}}_{i,p,y}\times {{\rm{A}}}_{i,j,y}\right)$, at county *j* located in province *p*, in year *y*. FC_*j, y*_ is the phosphorus/component fertilizer consumption statistics at county *j*, in year *y*; FC_*i, j, y*_ is the phosphorus/component fertilizer consumption estimates for crop type *i*, at county *j*, in year *y*. FR_*i, j, y*_ is phosphorus /component fertilizer rate for crop type *i*, at county *j*, in year *y*

#### Summation of phosphorus rate from phosphorus /component fertilizer rate

In the step, we transferred the above phosphorus /component fertilizer rate to phosphorus rate (i.e. P_2_O_5_ g/m^2^). The raw statistics of phosphorus fertilizer are already the amount of P_2_O_5_ from phosphorus fertilizer. For the data of component fertilizer, we converted it to grams of P_2_O_5_ by multiplying the weighted P_2_O_5_ content of imported and domestic-produced component fertilizer each year (Table 2). These steps allow us estimating the crop-specific phosphorus rate at county-level in the period of 2004**–**2016 by summing up the gram of P_2_O_5_ per unit of crop growing areas from both phosphorus and component fertilizers each year.

**Table 2:** The percentage of imported and domestic-produced component fertilizers over 2004–2016.

Year	Imported component fertilizer^1^ (%)	Domestic-produced component fertilizer^1^(%)
2004	36.0	64.0
2005	31.0	69.0
2006	24.5	75.5
2007	12.6	87.4
2008	4.6	95.4
2009	10.2	89.8
2010	8.3	91.7
2011	5.9	94.1
2012	7.4	92.6
2013	7.6	92.4
2014	6.3	93.7
2015	7.1	92.9
2016	5.3	94.7

^1^Statistics from Department of Rural Social and Economic Investigation^52–64^. The phosphorus content of imported component fertilizer is 60.0%, and the value of domestic-produced component fertilizer is 30.8% based on Lin and Li^65^.

#### Gridded maps of crop-specific phosphorus rate

As the final step, gridded crop growing area distribution maps of rice, wheat and maize were used as the mask raster, and extracted the pixels with the crop growing to produce the gridded maps of phosphorus rate for each crop in each year.

## Data Records

**Fig. 2:**
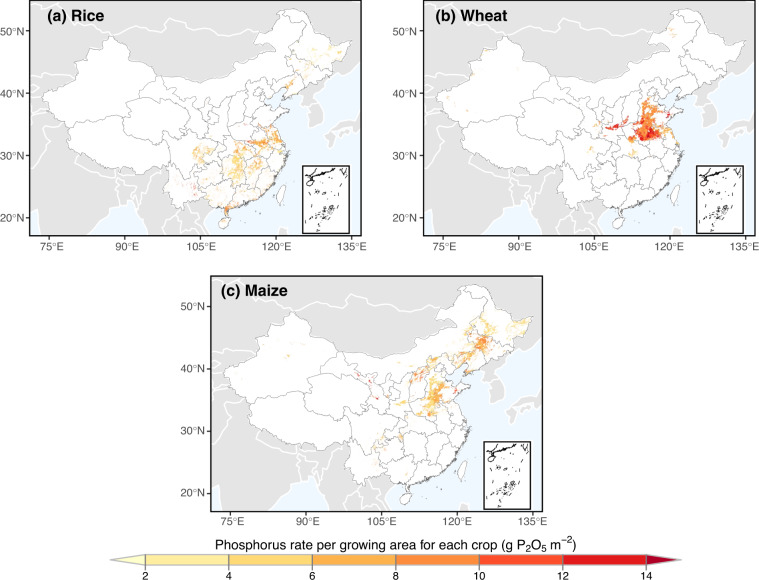
Spatial distribution of the crop-specific phosphorus rate averaged over 2004–2016. (**a**) map of rice; (**b**) map of wheat; (**c**) map of maize.

The CN-P^43^ dataset is publicly available for download from the Zenodo repository. The dataset is saved in the form of GeoTiff format. It is organized in folders according to crops, and named by the format of “CNP_<crop>_<year>.tif” (Table 3). Each file contains a 1 km gridded phosphorus rate map of rice, wheat and maize of China in certain year, with the unit grams of P_2_O_5_ per unit growing area (i.e. g P_2_O_5_ m^−2^). These files were saved with the WGS84 geographic coordinate system. As an example of CN-P, Fig. 2 demonstrates the spatial distribution of phosphorus rate for rice, wheat and maize averaged over 2004–2016. Table 4 gives the national average and time trend for each crop calculated based on CN-P. Wheat received the most phosphorus rate (8.7 g P_2_O_5_ m^−2^, *p* < 0.05), and maize showed the rapidest increasing trend (2.36% yr^−1^, *p* < 0.001) (Table 4).

**Table 3:** Nomenclature of data files for each crop for each year.

Crops	Year	Examples
Rice	2004–2016	CNP_rice_2013.tif
Wheat	2004–2016	CNP_wheat_2013.tif
Maize	2004–2016	CNP_maize_2013.tif

**Table 4:** The average and trends of crop-specific phosphorus rate in China over 2004–2016.

Crops	Phosphorus rate	Trends of phosphorus rate
	(g P_2_O_5_ m^−2^)	(% yr^−1^)
Rice^1^	6.5 b^3^	1.19 (*p* = 0.0054)^4^
Wheat^2^	8.7 a	0.59 (*p* = 0.389)
Maize	6.4 b	2.36 (*p* = 3.73e-06)

^1^The value here indicates the results for the national-level rice weighted by the single and double season rice growing areas.

^2^The value here indicates the results for the national-level wheat weighted by the spring and winter wheat growing areas.

^3^The significance between crops is based on Tukey HSD test. Different letter indicates statistical significance at *p* < 0.05.

^4^The time trend significant for each crop is based on *t*-test. The *p*-value in parentheses indicates the significance of time trends.

## Technical Validation

### Comparison with farmer’s survey by crops

**Fig. 3:**
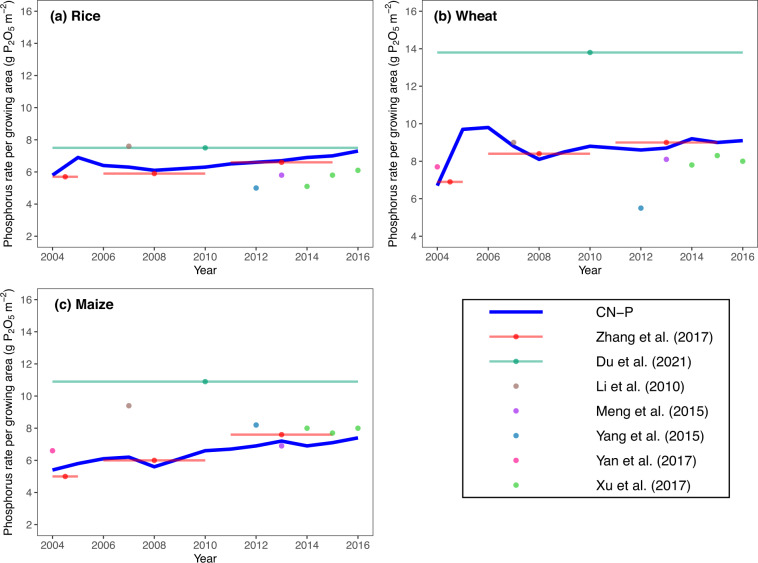
Comparison of crop-specific phosphorus rate with farmer’s surveys over 2004–2016. Blue line is the estimate of CN-P; points are estimates in individual years, and the collinear points are the estimates averaged by multiple years in farmer’s surveys.

To test the validity of CN-P, we compared the data with the estimates of farmer’s surveys^44–50^. As these estimates were conducted by questionnaire approach in individual years (denoted by points in Fig. 3) or average value over a period (denoted by collinear points in Fig. 3), these surveys could be viewed as an independent estimate. There was a large discrepancy between surveys, such as Du *et al*.^45^ estimated a much higher phosphorus rate especially for wheat (Fig. 3b) and maize (Fig. 3c) than others. In generally, the phosphorus rate of the three crops we estimated fell within the range of these studies (Fig. 3). For time trends, the farmer’s surveys also presented a more significant increasing trends for maize (Fig. 3c) compared with rice (Fig. 3a) and wheat (Fig. 3b), which is consistent with the trends of CN-P. This part of comparison suggests CN-P is comparable with these independent farmer’s surveys on phosphorus rate for each crop.

### Comparison with existing phosphorus datasets

Because there is no previous crop-specific phosphorus dataset, we calculated the total phosphorus consumption of China and spatial distribution of total phosphorus rate per cropland based on county statistics, which were used to compared with previous phosphorus datasets. In this analysis, as our county statistics include the total phosphorus and cropland data, our results based on county statistics should be viewed as observations.

**Fig. 4:**
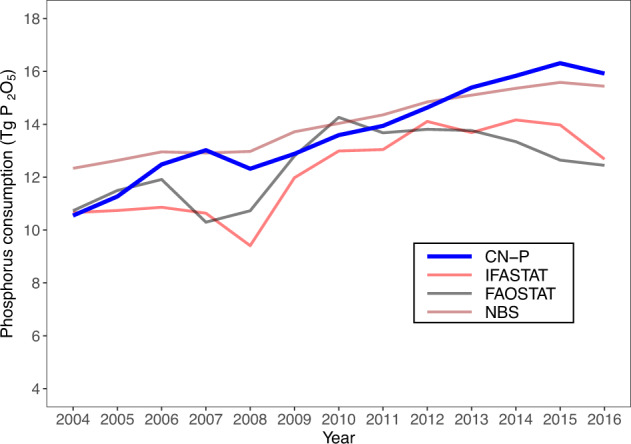
Comparison of phosphorus consumption derived from CN-P, IFASTAT, FAOSTAT and NBS over 2004–2016.

Firstly, we calculated the total phosphorus consumption based on county statistics and compared with the results of FAOSTAT^17^, IFASTAT^13^ and NBS^28^ in Fig. 4. The phosphorus fertilizer results varied between data sources, which reflects uncertainties in phosphorus fertilizer statistics even for these official data sources (Fig. 4). This is because of different survey method used by these databases. For example, IFASTAT database is based on the survey sent to country correspondents, including fertilizer associations, fertilizer companies, consultants, experts etc. But the FAOSTAT database is based on the FAOSTAT fertilizers questionnaire. In generally, our estimates were within the range of the three data sources before 2013. Beyond 2013, our estimates were higher than the other three databases, but close to the results of NBS (Fig. 4). Based on our results, there was an increase in the phosphorus consumption over time until 2015 and then followed by a slight drop after the year. The time trend pattern of our estimates is consistent with NBS (Fig. 4). The year of the changing point is the same with the year when China’s Ministry of Agriculture introduced the Action that seek to achieve zero growth in the use of chemical fertilizer in 2015^51^.

**Fig. 5:**
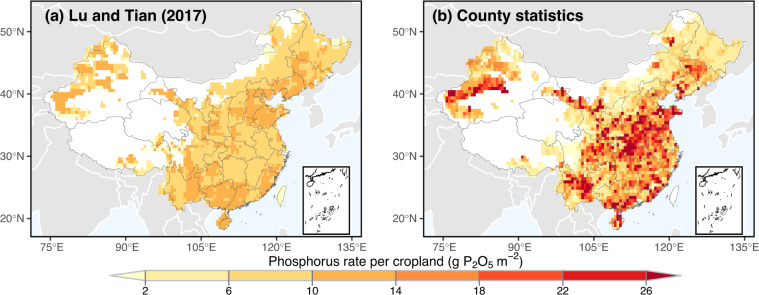
Comparison of average phosphorus rate per cropland over 2004–2013. (**a**) maps of Lu and Tian^14^; (**b**) maps based on county statistics. To compare them, the same mask raster was used.

Secondly, we further compared the spatial distribution map of total agricultural phosphorus rate per cropland with Lu and Tian^14^ averaged over 2004–2013 (Note: the latest year reported in Lu and Tian^14^ is 2013). The definition of cropland here is different from crop growing area used in CN-P. Cropland could be used to grow more than one crop seasons in the same field. Using our county statistics, we calculated the total agricultural phosphorus rate per cropland. For Lu and Tian’s^14^ data, we converted grams of phosphorus in their dataset to grams of P_2_O_5_ by multiplying by the ratio of 142/62. We found that the spatial heterogeneity differed between the two datasets (Fig. 5). County statistic is higher than the results estimated by Lu and Tian^14^. Using county statistics, some regions reached to 18–26 g P_2_O_5_ per square meter cropland (Fig. 5b), while most areas were less than 18 g P_2_O_5_/m^2^ in the dataset of Lu and Tian^14^ (Fig. 5a). Another disagreement is that there was a high phosphorus rate hotspot in the central of China presented by our county statistics (Fig. 5b), while this was not shown in Lu and Tian^14^ (Fig. 5a). This reflects the data construction approach of Lu and Tian^14^, which multiplied a national-level phosphorus rate with gridded cropland area and adjusted with the national data of IFASTAT inventory. Such approach will lead to the underestimation of phosphorus rate because the national level phosphorus rate could smooth out the variability in the sub-national scale and underestimate the hotspot within country. Therefore, the presence of spatial heterogeneity of Lu and Tian^14^ is majorly from the gridded cropland areas, and cannot reflect the actual spatial heterogeneities. This part of analysis suggests that introducing county-level statistics is critically needed to show an improved estimates and spatial distribution in phosphorus rate.

### Uncertainty and future needs

The uncertainties of CN-P are mainly from the following aspects: (1) We applied the provincial crop-specific phosphorus consumption ratio at county scales. Currently, there is no available statistics on county-level phosphorus consumption for each crop in China, and it is unlikely that such data will ever become available in a near future. (2) For conversing component fertilizer to phosphorus contents, we used a static phosphorus content in each year for the whole China (Table 2). Map accuracy can be further improved if the crop-specific county and phosphorus ratio of component fertilizer information are available. (3) We note that data construction before the year of 2004 is quite challenge as the “Cost-benefit Statistics of Agricultural Products” do not record crop-specific phosphorus fertilizer before 2004 and only document the data of total fertilizer (i.e. the sum of nitrogen, phosphorus and potassium fertilizers). If we could collect longer-term datasets, or more accurate approaches to gap-fill our data, we will provide future improvements. Therefore, a continuous survey of crop-specific fertilizer data and development of dynamic crop-type maps to meet the needs of current study are imminently required. The longer time series of crop-specific phosphorus rate maps will improve the characterization of geospatial and temporal patterns of phosphorus fertilizer management in China.

## Data Availability

The code of CN-P is archived at the Zenodo repository: 10.5281/zenodo.7460564.
